# Value‐based payment models and management of newly diagnosed prostate cancer

**DOI:** 10.1002/cam4.6810

**Published:** 2023-12-26

**Authors:** Avinash Maganty, Samuel R. Kaufman, Mary K. Oerline, Kassem S. Faraj, Megan E. V. Caram, Vahakn B. Shahinian, Brent K. Hollenbeck

**Affiliations:** ^1^ Division of Health Services Research, Department of Urology University of Michigan Ann Arbor Michigan USA; ^2^ Division of Hematology/Oncology, Department of Internal Medicine University of Michigan Ann Arbor Michigan USA; ^3^ VA Health Services Research & Development, Center for Clinical Management Research VA Ann Arbor Healthcare System Ann Arbor Michigan USA; ^4^ Division of Nephrology, Department of Internal Medicine University of Michigan Ann Arbor Michigan USA

**Keywords:** Medicare, prostate cancer, quality of care, value‐based payment

## Abstract

**Objective:**

To examine the effect of urologist participation in value‐based payment models on the initial management of men with newly diagnosed prostate cancer.

**Methods:**

Medicare beneficiaries with prostate cancer diagnosed between 2017 and 2019, with 1 year of follow‐up, were assigned to their primary urologist, each of whom was then aligned to a value‐based payment model (the merit‐based incentive payment system [MIPS], accountable care organization [ACO] without financial risk, and ACO with risk). Multivariable mixed‐effects logistic regression was used to measure the association between payment model participation and treatment of prostate cancer. Additional models estimated the effects of payment model participation on use of treatment in men with very high risk (i.e., >75%) of non‐cancer mortality within 10 years of diagnosis (i.e., a group of men for whom treatment is generally not recommended) and price‐standardized prostate cancer spending in the 12 months after diagnosis.

**Results:**

Treatment did not vary by payment model, both overall (MIPS—67% [95% CI 66%–68%], ACOs without risk—66% [95% CI 66%–68%], ACOs with risk—66% [95% CI 64%–68%]). Similarly, treatment did not vary among men with very high risk of non‐cancer mortality by payment model (MIPS—52% [95% CI 50%–55%], ACOs without risk—52% [95% CI 50%–55%], ACOs with risk—51% [95% CI 45%–56%]). Adjusted spending was similar across payment models (MIPS—$16,501 [95% CI $16,222–$16,780], ACOs without risk—$16,140 [95% CI $15,852–$16,429], ACOs with risk—$16,117 [95% CI $15,585–$16,649]).

**Conclusions:**

How urologists participate in value‐based payment models is not associated with treatment, potential overtreatment, and prostate cancer spending in men with newly diagnosed disease.

## 1 INTRODUCTION

Prostate cancer is among the most common and expensive malignancies in the United States, with 288,300 cases annually and spending approaching $22 billion.^1^, ^2^ Moreover, not all men benefit from local treatment, particularly those with non‐aggressive disease or a high likelihood of death from competing risks.^3^ Nonetheless, financial incentives in fee‐for‐service may spur treatment in such discretionary circumstances, manifesting as overtreatment and unnecessary spending.^4^, ^5^, ^6^ Payment reforms outlined by the Medicare Access and CHIP Reauthorization Act (MACRA) shift the focus of the delivery system from volume to value. By aligning payment with quality, this policy has the potential to mitigate financial incentives in fee‐for‐service.

As part of MACRA, physicians participating in Medicare are required to participate in a value‐based payment model beginning in 2017. The models most relevant to the urologist are the merit‐based incentive payment system (MIPS) and accountable care organizations (ACOs) with and without financial risk.^7^, ^8^ On one hand, the extent to which these models may influence prostate cancer care is likely to be driven by the magnitude of the financial risk to which physicians are exposed. For example, in contrast to those participating in ACOs without risk, urologists in ACOs with risk are responsible for repaying a percentage (40%–75%) of excess spending, which may mitigate low value care (e.g., treating men with prostate cancer at high risk of death from competing risks).^9^ In contrast, excess spending in the MIPS program is not directly penalized. Rather, spending represents only one of four components by which physicians are scored. Further, the majority of physicians achieve nearly perfect scores in MIPS, with very few exposed to financial penalties.^10^, ^11^, ^12^ On the other hand, the models may not differentially affect quality because motivations to reduce low value utilization for specialty care may be offset by the financial incentive for volume in Medicare's fee‐for‐service system in which the models are embedded. The extent to which participation in these models affects care for a specialty condition, such as prostate cancer, is unclear.

For this reason, we performed a national study of Medicare beneficiaries with newly diagnosed prostate cancer to determine how urologist participation in value‐based payment models following MACRA implementation affects use of treatment, potential overtreatment, and spending. We hypothesize, due to direct liability in ACOs with financial risk to constrain spending, participants will be more likely to constrain utilization, thereby leading to lower potential overtreatment and spending compared to urologists participating in MIPS or ACOs without financial risk.

## 2 METHODS

### 2.1 Data and study population

We performed a retrospective cohort study of men with newly diagnosed prostate cancer between 2017 and 2019 using a 20% random sample of national Medicare claims. All men had at least 1 year of follow‐up, with data available through December 31, 2020. We included men who received a new diagnosis of prostate cancer as identified using a previously validated algorithm with 99.8% specificity and 88.7% positive predictive value.^13^ Briefly, we identified men with at least two Evaluation and Management visit codes for a diagnosis of prostate cancer and who had undergone a prostate biopsy within 180 days of the first visit code. Men with a claim in the preceding 12‐month period that was associated with a diagnosis of prostate cancer were excluded. Additionally, men who were not continuously enrolled in Medicare Parts A and B 1 year before and 1 year after diagnosis as well as those participating in managed care plans were excluded from the study, given the payment models only apply to beneficiaries in fee‐for‐service.

All men were assigned to their primary urologist using established methods.^4^ Briefly, each patient was assigned to the urologist under whom the majority (>75%) of Evaluation and Management visit claims were submitted. Urologists were then assigned to their respective practices using their tax identification number under which the plurality of their claims were submitted.^14^ Practices were further categorized by their organization—either single specialty urology practices or multispecialty groups—based on prior work.^15^

The primary exposure was the value‐based model to which the urologist was aligned. Unless participating in another payment model, physicians are subject to the MIPS model by default unless they do not meet Medicare's participation criteria (i.e., submit more than $90,000 for Part B professional services, see more than 200 Part B patients, and provide more than 200 covered professional services).^8^ Urologists were aligned to MIPS if their NPI was identified in Medicare's Quality Payment Program File and had a MIPS score for a given performance year.^16^ Urologists were aligned to an ACO if their NPI was listed in the Medicare Shared Savings Program ACO Provider‐level file for a given year.^17^ ACOs were further categorized as those with and without risk using the publicly available Medicare ACO Participants file.^18^ We verified alignment of the physicians that were not allocated to a model using the above methodology by manually searching all physicians by their NPI using Medicare's Quality Payment Program eligibility website, which provides information on Medicare payment model participation by year.^8^ We excluded urologists who were not aligned to any model (14%), due to reasons above, from the analysis. This resulted in three groups for comparison: Men managed by physicians aligned to MIPS, ACOs without risk, and ACOs with risk.

### 2.2 Outcomes

The primary outcome was use of any local treatment for prostate cancer within 12 months of diagnosis. Men were considered to have received treatment if they underwent either surgery, radiation, brachytherapy, or cryotherapy within 12 months of diagnosis. Treatment modalities were identified using the Carrier and Outpatient files based on Healthcare Common Procedure Coding System Codes. Men who did not undergo local treatment within 12 months of diagnosis were classified as undergoing conservative management, which is inclusive of both active surveillance, watchful waiting, or androgen deprivation. Hypothesizing that incentives to improve quality and reduce spending may lead to a reduction in low value treatment, we also assessed treatment among men with greater than 75% likelihood of non‐cancer mortality within 10 years of diagnosis. This represents a group of men who are least likely to benefit from local treatment due to a combination of older age and significant medical conditions.^19^, ^20^ Using a claims‐based model with high discrimination (*c* = 0.82) from prior work,^4^ we estimated the risk of non‐cancer death within 10 years for each beneficiary and identified those with greater than 75% risk. Importantly, the purpose for using this model was not to predict non‐cancer mortality precisely, but rather to identify a group of men least likely to benefit from treatment due to age and comorbid conditions. Furthermore, this model has demonstrated construct validity, differentiating how patients are managed, suggesting it tracks with important factors clinicians may use to aid decision making.^21^, ^22^, ^23^

Our secondary outcome was spending per beneficiary for prostate cancer services in the first 12 months after diagnosis, which was assessed among all beneficiaries with a new prostate cancer diagnosis and by treatment modality (i.e., surgery and radiation). Spending was defined as total price‐standardized payments for prostate cancer services for the 12‐month period following diagnosis. Standardized payments were used to control for geographic variation in payments.

### 2.3 Analysis

To understand engagement of urologists in each of the payment models over time, we examined the distribution of urologists aligned to value models before (2014–2016) and after MACRA initiation (2017–2019).

We then compared demographic and clinical characteristics of men with a new diagnosis of prostate cancer between 2017 and 2019 across the three groups of value‐based payment models: MIPS, ACO with risk, ACO without risk. Differences between categorical variables were assessed using the chi‐squared test.

We fit multivariable mixed‐effects models with a logit link and a random intercept for the urologist to estimate patient‐level use of treatment. Models were adjusted for age, race, socioeconomic status at the zip‐code level,^24^ comorbidity,^25^ rural residence, year of diagnosis (to adjust for secular trends), and practice organization. Models were also adjusted for market characteristics at the zip‐code level including urologist density, radiation oncologist density, number of hospital beds, and Medicare Advantage penetration. The last serves as a proxy for the degree of familiarity with fee‐for‐service value‐based models in each market. Because the way in which physicians organize themselves (i.e., single specialty vs. multispecialty group) affects the type of payment model they may engage with and is associated with use of treatment,^6^ we also adjusted for practice organization. To assess potential overtreatment, this approach was repeated using the cohort of men who had greater than 75% risk of non‐cancer mortality within 10 years of diagnosis. Predicted probabilities were obtained using Stata's margins command.

To estimate differences in per beneficiary spending for prostate cancer care in the 12 months after diagnosis, a similar modeling approach was used. However, instead of a logit link, a log link with a gamma distribution was used because the distribution of payments was skewed.

Because the ability to meet financial benchmarks imposed by value‐based payment models may differ based on the organizational context of physician practices (i.e., single specialty vs. multispecialty groups), we decided a priori to perform a separate analysis to determine whether the effects of value‐based payment models differed between single specialty and multispecialty urology groups (inclusive of those affiliated with a hospital). For instance, compared to single specialty practices, multispecialty groups may be more effective in reducing spending for specialty care by leveraging a more robust infrastructure to track quality and coordinate care with primary care physicians.^26^ In order to determine whether there is a differential effect, we repeated the main analysis using an interaction term between value‐based payment model and practice organization of the managing urologist.

We also performed several sensitivity analyses to confirm the robustness of our findings. First, Medicare issued a final rule, referred to as “Pathways to Success,” which allowed physicians to drop out of ACOs or transition between ACOs with and without financial risk starting July 1, 2019. To mitigate potential bias from these transitions in 2019, we repeated our analysis after excluding men cared for by urologists who transitioned between, or out of, ACOs in 2019. Second, we ascertained value‐based payment model participation for urologists not identified in either the quality payment program files (for MIPS) or the ACO provider files (12%) by querying Medicare's quality payment program site using the physician's NPI. Because this site is primarily a technical resource, the payment model designations may not be accurate. We therefore repeated the primary analysis after excluding men (6%) who were cared for by urologists whose payment model participation was identified using the quality payment program website. The results of the sensitivity analyses were consistent with our main findings, and therefore, we only present the latter.

All analyses were performed using SAS 9.4 (Cary, North Carolina) and Stata 17 (College Station, Texas). All tests were two‐sided with a type 1 error set at 0.05. The study was deemed exempt from review by the institutional review board.

## 3 RESULTS

**FIGURE 1:**
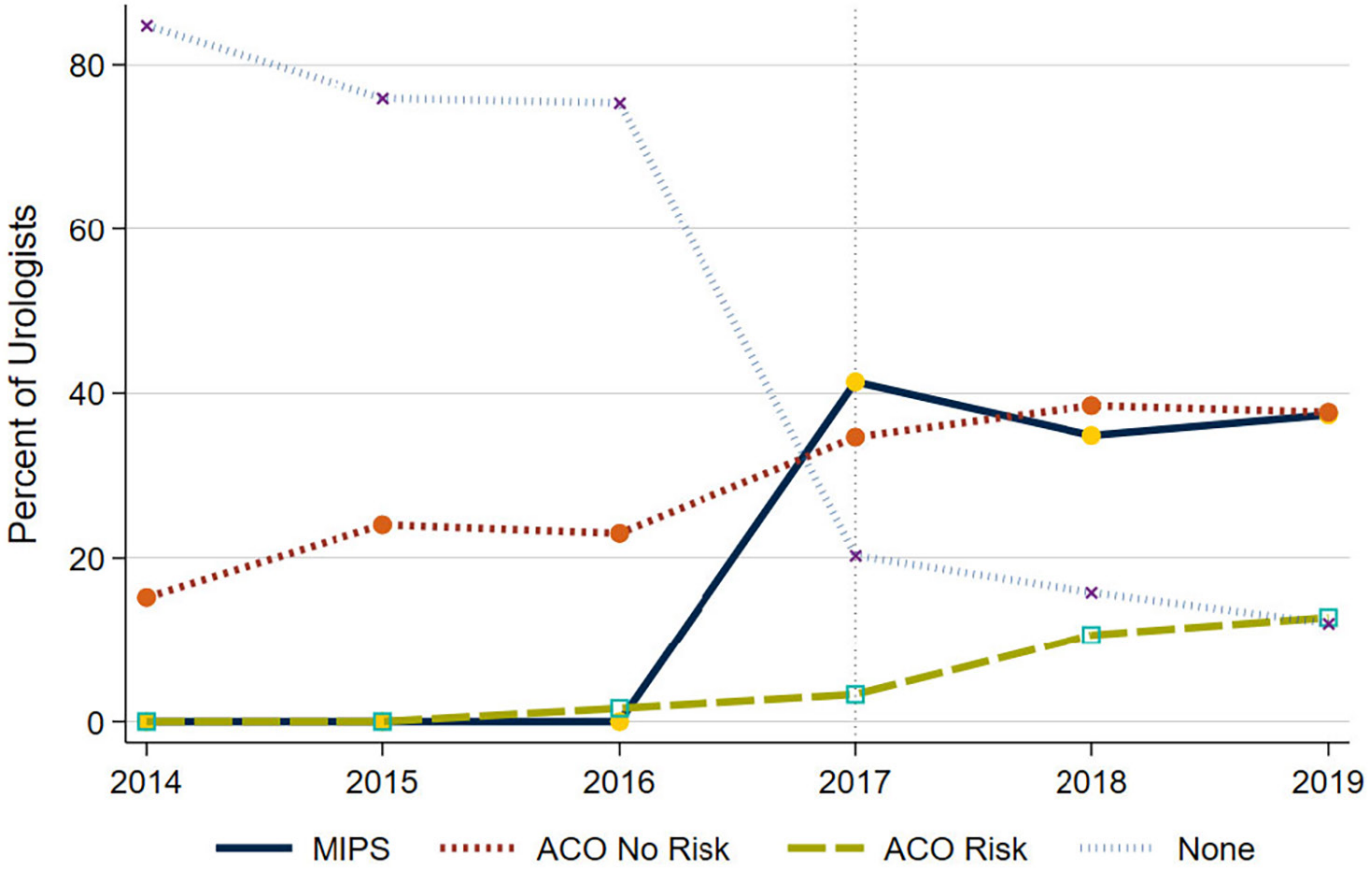
Percent of urologists participating value‐based payment models before and after MACRA initiation in 2017 (indicated by dotted line).

Prior to MACRA initiation in 2017, most urologists were not aligned to a value‐based payment model (e.g., ACOs); however, alignment increased over time. Specifically, from 2014 to 2016, alignment to ACOs without risk increased from 15% to 23% (*p* < 0.001 for trend). Few urologists were aligned to ACO models with risk, although this increased with time (<1% in 2013 and 2% in 2016, *p* < 0.001 for trend). After MACRA initiation in 2017, 42% of urologists were aligned to MIPS, and this declined to 37% in 2019 (*p* < 0.001 for trend). Conversely, alignment increased for ACOs with risk (3% in 2017 to 13% in 2019, *p* < 0.001 for trend) and for ACOs without risk (35% in 2017 to 38% in 2019, *p* < 0.001 for trend) (Figure 1).

We identified 30,964 men diagnosed with localized prostate cancer between 2017 and 2019 with follow‐up through 2020. Patient and market characteristics are illustrated by value‐based payment model alignment of the managing urologist in Table 1. Demographic and clinical characteristics varied by payment model. Although most of these differences were modest, men managed by urologists aligned to MIPS were more likely to be from racial minorities and reside in urban areas, compared to those managed by urologists aligned to ACOs. Furthermore, MIPS participating urologists were more likely to be in smaller practices compared to those aligned to ACOs, who were more likely to be part of multispecialty groups.

**TABLE 1:** Cohort characteristics, stratified by payment model of primary urologists.

*N*	MIPS	ACO no risk	ACO risk	*p*‐Value
	14,376	12,937	3653	
Age categories				
66–69	4399 (30.6%)	4316 (33.4%)	1218 (33.3%)	0.001
70–74	5180 (36%)	4386 (33.9%)	1320 (36.1%)	
75–79	3082 (21.4%)	2742 (21.2%)	736 (20.1%)	
80–84	1255 (8.7%)	1096 (8.5%)	275 (7.5%)	
>85	460 (3.2%)	397 (3.1%)	104 (2.8%)	
Comorbidity score				
0	7914 (55.1%)	7296 (56.4%)	2069 (56.6%)	0.030
1	2595 (18.1%)	2350 (18.2%)	615 (16.8%)	
2	1999 (13.9%)	1764 (13.6%)	514 (14.1%)	
3 or more	1868 (13%)	1527 (11.8%)	455 (12.5%)	
Socioeconomic status				
Low	4256 (29.6%)	3808 (29.4%)	933 (25.5%)	<0.001
Middle	4798 (33.4%)	4805 (37.1%)	1289 (35.3%)	
High	5322 (37%)	4324 (33.4%)	1431 (39.2%)	
Rural residence	2198 (15.4%)	3223 (25%)	628 (17.2%)	<0.001
Race				
White	11,808 (82.5%)	11,061 (85.6%)	3104 (85.1%)	<0.001
Black	1347 (9.4%)	938 (7.3%)	279 (7.7%)	
Other	1160 (8.1%)	918 (7.1%)	263 (7.2%)	
Practice organization				
Small	2366 (16.5%)	1100 (8.5%)	270 (7.4%)	<0.001
Medium	3070 (21.4%)	2552 (19.7%)	582 (15.9%)	
Large	4645 (32.3%)	2205 (17%)	914 (25%)	
MSG	3499 (24.4%)	5956 (46%)	1629 (44.6%)	
Hospital	786 (5.5%)	1121 (8.7%)	258 (7.1%)	
Urologists per 100K				
Low (<53)	7139 (49.9%)	6860 (53.1%)	1586 (43.5%)	<0.001
Intermediate	4074 (28.5%)	3251 (25.2%)	1167 (32%)	
High (>87)	3102 (21.7%)	2806 (21.7%)	893 (24.5%)	
Radiation oncologists per 100K				
Low (<22)	7335 (51.2%)	6836 (52.9%)	1634 (44.8%)	<0.001
Intermediate	3601 (25.2%)	2879 (22.3%)	963 (26.4%)	
High (>37)	3379 (23.6%)	3202 (24.8%)	1049 (28.8%)	
Hospital Beds per 100K				
Low (<4750)	7425 (51.9%)	6282 (48.6%)	1737 (47.6%)	<0.001
Intermediate	3495 (24.4%)	2856 (22.1%)	888 (24.4%)	
High (>6854)	3394 (23.7%)	3779 (29.3%)	1021 (28%)	
Medicare managed care penetration				
Low (<5%)	700 (4.9%)	865 (6.7%)	140 (3.8%)	<0.001
Intermediate	4876 (34.2%)	5031 (39%)	1099 (30.2%)	
High (>16.2%)	8694 (60.9%)	7006 (54.3%)	2405 (66%)	

Abbreviations: ACO, accountable care organization; MIPS, merit‐based incentive payment system.

**FIGURE 2:**
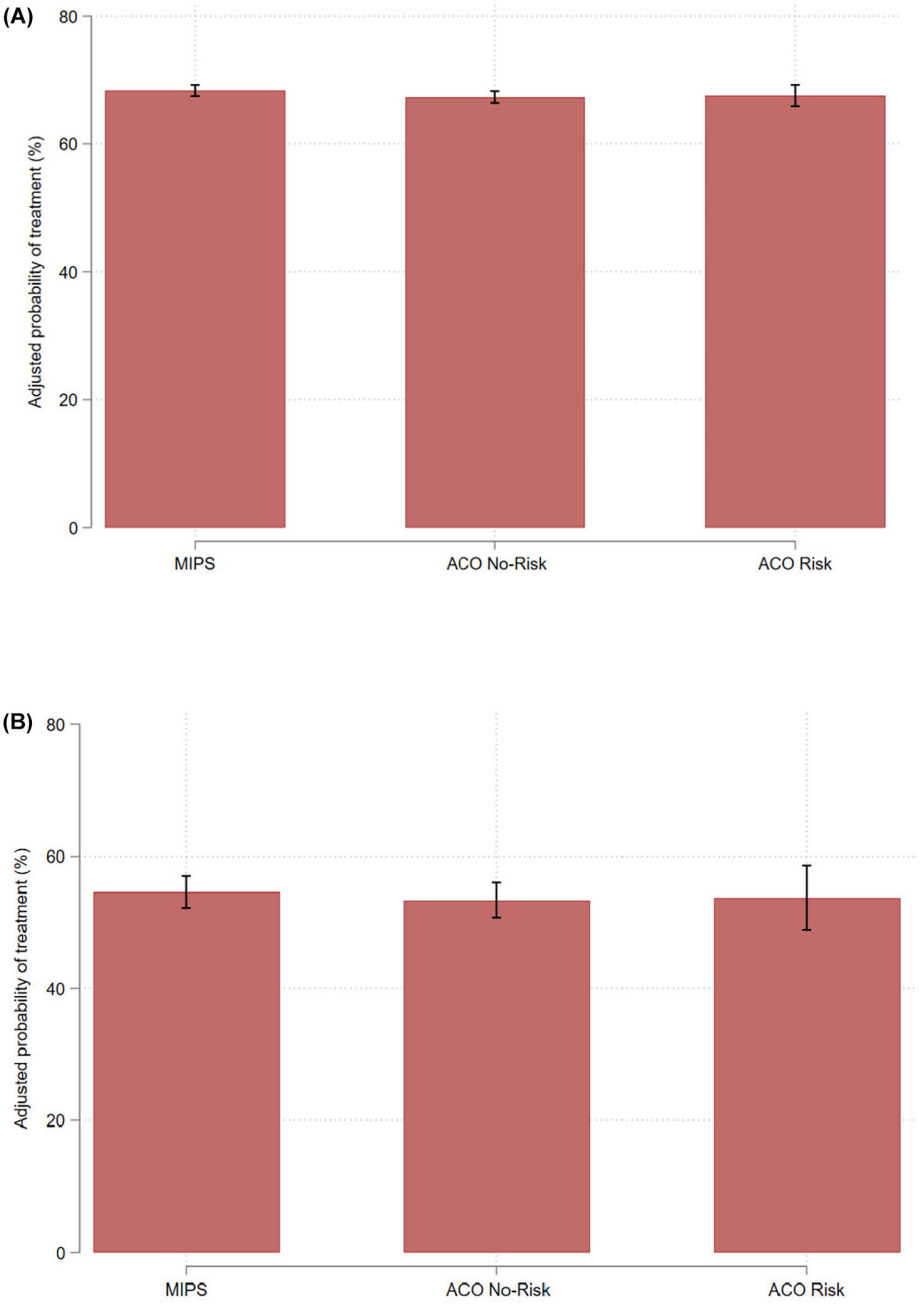
Adjusted percent of (A) all men with newly diagnosed prostate cancer receiving treatment and (B) those with greater than 75% risk of non‐cancer mortality within 10 years, stratified by payment model of urologist. Models adjusted for age, comorbidity, socioeconomic status, race, rural residence, practice organization, year of diagnosis, urologist density, radiation oncologist density, number of hospital beds per 100K residents, and Medicare advantage penetration.

The use of treatment did not vary by value‐based payment model, as shown in Figure 2A (MIPS—67%, 95% CI 66%–68%, ACOs without risk—66% [95% CI 66%–68%], ACOs with risk—66% [95% CI 64%–68%]). Similarly, as shown in Figure 2B, there was no difference in the likelihood of receiving treatment among men with greater than 75% risk of non‐cancer mortality by the value‐based payment model to which the urologist was aligned (MIPS—52% [95% CI 50%–55%], ACOS without risk—52% [95% CI 50%–55%], ACOS with risk—51% [95% CI 45%–56%]).

Adjusted spending per beneficiary for the 12‐month period after diagnosis did not vary by payment model of the urologist (MIPS—16,501 [95% CI $16,222–$16,780], ACOs without risk—$16,140 [95% CI $15,852–$16,429], ACOs with risk—16,117 [95% CI $15,585–$16,649]). Similarly, spending did not vary among men who received treatment (MIPS—$22,128 [95% CI $22,845–$22,140], ACOs without risk—$21,829 [95% CI $21,533–$22,124], ACOs with risk $21,572 [95% CI $21,050–$22,095]), nor by treatment modality (Table 2).

**TABLE 2:** Adjusted spending ($) for prostate cancer services per beneficiary in the 12‐month period after diagnosis by payment model.

	Value‐based model		
	MIPS	ACO without risk	ACO with risk
All beneficiaries	16,501 (16,222, 16,780)	16,140 (15,852, 16,429)	16,117 (15,585, 16,649)
Treated beneficiaries	22,128 (21,845, 22,410)	21,829 (21,533, 22,124)	21,572 (21,050, 22,095)
Surgery	15,619 (15,247 15,991)	15,577 (15,215, 15,938)	15,784 (15,218, 16,441)
Radiation	24,918 (24,580, 25,256)	25,025 (24,657, 25,393)	24,746 (21,088, 25,405)

*Note*: Models adjusted for age, comorbidity, socioeconomic status, race, rural residence, practice organization, year of diagnosis, urologist density, radiation oncologist density, number of hospital beds per 100K residents, and Medicare advantage penetration.

Abbreviations: ACO, accountable care organization; MIPS, merit‐based incentive payment system.

**FIGURE 3:**
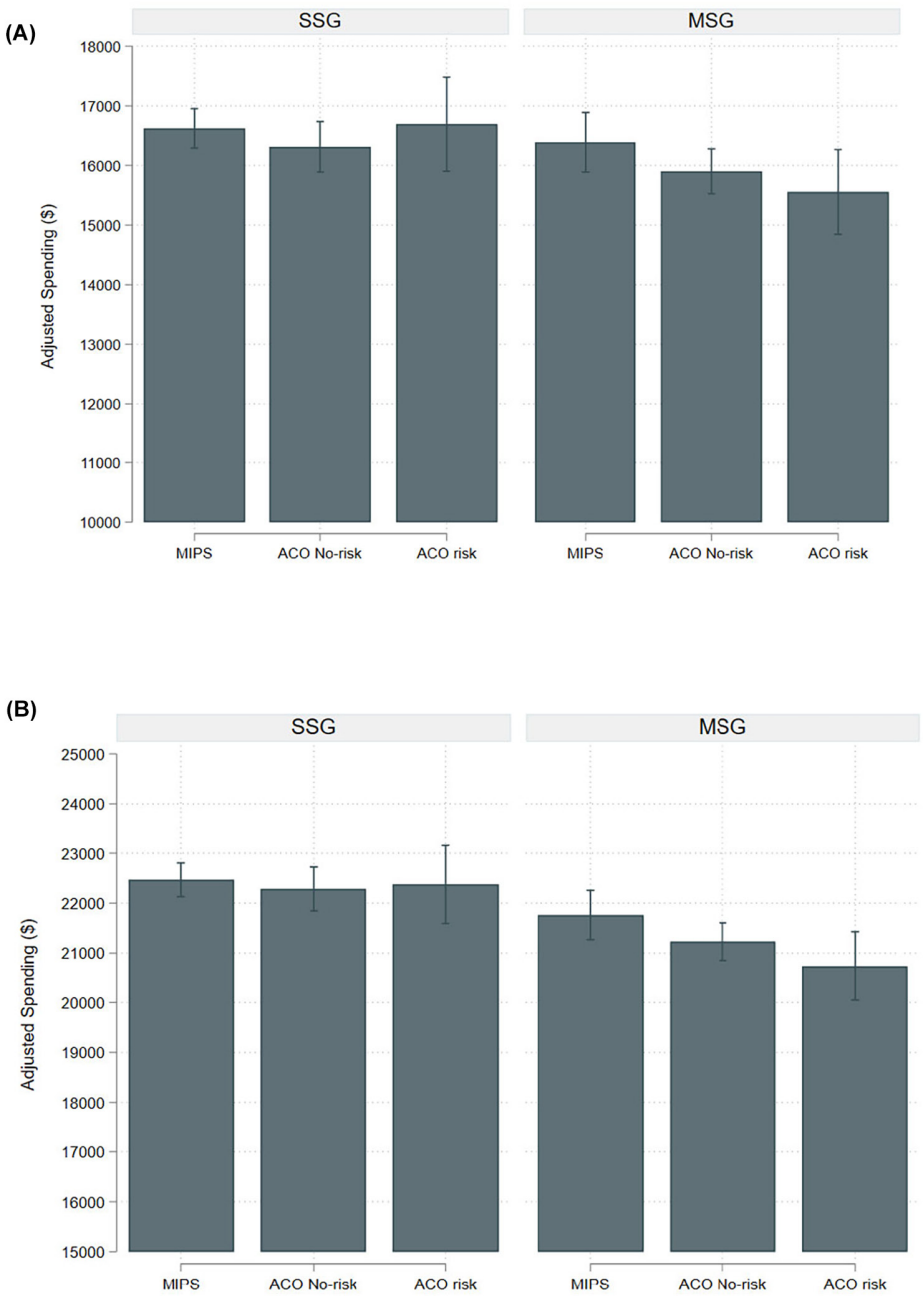
Adjusted spending for prostate cancer services per beneficiary in the 12‐month period after diagnosis by payment model among (A) all beneficiaries and (B) those who received treatment, stratified by single specialty and multispecialty urology groups. Models adjusted for age, comorbidity, socioeconomic status, race, rural residence, practice organization, year of diagnosis, urologist density, radiation oncologist density, number of hospital beds per 100K residents, and Medicare advantage penetration. SSG: single specialty group; MSG: multispecialty group.

The effect of value‐based payment model participation on use of treatment and potential overtreatment was similar between urologists in single specialty groups compared to those in multispecialty groups. Additionally, spending for all beneficiaries by payment model did not differ by practice organization (Figure 3A). For men who received treatment and were managed by urologists in multispecialty groups, spending was lower for those managed by urologists aligned to ACOs with risk compared to those managed by urologists aligned to MIPS (ACOs with risk—$21,761 [95% CI $21,267–$22,254], MIPS—$20,737 [95% CI $20,048–$21,427], Figure 3B).

## 4 DISCUSSION

Physician payment reform initiated by MACRA aims to increase the value of healthcare delivered by improving quality and reducing spending.^27^ By aligning of financial incentives with quality, payment reform has the potential to mitigate the use of treatment of prostate cancer among men for whom the benefits are uncertain or do not outweigh the risks of therapy.^4^, ^5^ We found that men with newly diagnosed prostate cancer had similar rates of treatment, regardless of the payment model to which their primary urologist was aligned. This was also true for men with greater than 75% risk of non‐cancer mortality within 10 years, arguably a group who are least likely to benefit from treatment.^19^, ^20^ Furthermore, spending for the 12‐month period after diagnosis did not vary by payment model.

Although we hypothesized that the varying strength of incentives within payment models may differentially influence prostate cancer management, use of treatment did not differ by the model to which the urologist was aligned. The similar rates of treatment across payment models—even among men least likely to benefit—may be explained by several factors. First, the models are embedded in Medicare's fee‐for‐service system which may offset any incentives to constrain utilization, particularly for conditions such as prostate cancer, managed by surgical specialties. Second, most of the measures of quality tracked by the models are relevant for primary care conditions, which may limit their effectiveness in improving care for specialty conditions such as prostate cancer.^11^ Third, physicians may meet quality and spending benchmarks in the payment models through other mechanisms which do not impact use of treatment (i.e., improving care coordination, minimizing preventable hospitalizations, and reducing unnecessary testing).^28^, ^29^

In addition to similar rates of treatment across payment models, spending per beneficiary in the 12‐month period also did not vary by model, even when assessed by treatment modality.^30^ No prior study has examined the effects of MIPS—the model to which most physicians are aligned to by default—on spending. Nonetheless, criticisms have been raised regarding the model's ability to constrain utilization for several reasons.^11^ In particular, there is concern that penalties in MIPS can be avoided without meaningfully engaging in quality improvement, partially due to the physicians’ ability to self‐select the measures to report and preferentially choosing those in which they already excel.^31^ Therefore, incentives in MIPS to constrain use are likely weaker than those within ACO models, as spending represents a minor component (up to 30%) of how physicians performance is assessed within the program.^12^, ^32^ Financial rewards in ACOs, on the contrary, are almost completely dependent on their ability to remain below spending thresholds. Moreover, prior work has demonstrated reduction in spending for inpatient and outpatient hospital care among those aligned to ACOs compared to those who were not aligned.^33^ For these reasons, we expected spending to be less compared to those aligned to MIPS.^33^ However, we did not identify any differences in spending across payment models. Prior work examining the effect of ACOs on specialty conditions, including prostate cancer, has demonstrated mixed results.^34^, ^35^ This may be due, in part, to the limited engagement of specialists with ACOs early on, the few metrics applicable to specialty care, and the inability of incentives within ACOs to offset those within fee‐for‐service for procedural conditions.^36^ Alternatively, there may have been a movement towards judicious utilization following MACRA passage, motivating physicians to minimize spending, irrespective of the payment model to which they were aligned. Interestingly, among men managed by multispecialty groups, spending was lower for those aligned to ACOs with risk compared to those aligned to MIPS by $1024. Although this analysis was primarily exploratory, this finding is consistent with the notion that multispecialty groups are well positioned to meet the demands of ACOs with risk because of they are primary care centric and emphasize care coordination, quality measurement, and cost reduction.^26^ Further, primary care physicians, who are at the center of multispecialty groups, may have more control over the totality of care through management of referrals to specialists, which may help these practices contain costs.^37^ Importantly, this differential impact suggests that the influence of payment models and their ability to constrain low value utilization may vary by organizational context. A single model may not fit all practice types.

Our study must be interpreted in the context of several limitations. First, we cannot ascertain disease grade, stage (i.e., metastatic vs. non‐metastatic) which certainly motivates treatment and is required for assessment of appropriateness. However, the analysis is looking at large groups of patients and there is no rationale for why disease severity would vary by payment model and lead to differential use of treatment and spending across such large groups. Although other datasets, such as SEER‐Medicare, do offer measurement of disease severity, they are not nationally representative such as that used for this analysis. Second, there is likely a degree of random error introduced by our methodology of assigning a physician to a payment model. For instance, ACO participation was based on the urologist alignment and not the beneficiary or primary care provider. Additionally, some ACOs (those without financial risk) also report within MIPS, although the primary incentives are driven by the ACO model and not MIPS. This misclassification may attenuate the observed associations and thus lead to conservative estimates. Third, participation in value‐based payment models, particularly ACOs with risk, is likely to be influenced by the organization of the practice, with multispecialty groups being the best positioned to engage in these models. Practice organization may also be associated with treatment, based on prior work demonstrating varying use of conservative management by organization context.^6^ We attempted to address this confounding by adjusting for practice organization and performing a sensitivity analysis using only patients managed by multispecialty groups, which yielded similar findings. Fourth, we were unable to identify value‐based payment model alignment for 14% of urologists and were not included in the analysis.

These limitations notwithstanding, our findings have important implications. With ongoing concern for overtreatment of prostate cancer and rising costs of care, the mandatory participation in payment models initiated by MACRA has the potential to improve the value of care.^38^, ^39^ However, based on these findings, urologist engagement, with these models, at least in the manner in which they did during the study period, may not significantly influence management for a specialty condition like prostate cancer. Additionally, mandatory participation in these models may result in undue time and financial burden for physicians, without significantly improving patient care. Interestingly, the impact of payment models may differ by practice context, suggesting a one‐size‐fits all approach to value‐based payment may be ineffective, particularly for specialty care. Because value‐based payment models available to most specialists tend not to measure quality relevant to the conditions they treat, there may be increased focus on reducing costs—particularly for those participating in models with downside risk. This may inadvertently lead to sacrificing quality that is important to patients in attempt to reduce costs. Moving forward, condition‐specific alternative payment models for specialists may be more effective in improving quality and reducing costs. Alternatively, more cohesive integration of specialists into models such as ACOs could help achieve value for specialty conditions. For example, the Making Care Primary Model has been introduced by Medicare and mandates a relationship between primary care and specialty care partners with direct incentives for specialists providing high quality care.^40^

## 5 CONCLUSIONS

In conclusion, MIPS and ACOs with and without financial risk did not differentially affect treatment of Medicare beneficiaries with newly diagnosed prostate cancer. Importantly, we did not observe any differences in use of treatment among men least likely to benefit across value‐based models, suggesting that any constraints on utilization are insufficient to influence treatment in this group. Similarly, spending per beneficiary did not vary by payment model. As Medicare intends to transition both physicians and beneficiaries into care relationships accountable for both cost and quality by 2030, it will be important to understand the relationship between constraints on utilization and quality of care.

## AUTHOR CONTRIBUTIONS

**Avinash Maganty:** Conceptualization (equal); formal analysis (equal); methodology (equal); visualization (equal); writing – original draft (equal); writing – review and editing (equal). **Samuel R. Kaufman:** Data curation (equal); methodology (equal); writing – review and editing (equal). **Mary K. Oerline:** Data curation (equal); methodology (equal); writing – review and editing (equal). **Kassem S. Faraj:** Conceptualization (equal); investigation (equal); writing – review and editing (equal). **Megan E. V. Caram:** Investigation (equal); supervision (equal); writing – review and editing (equal). **Vahakn B. Shahinian:** Conceptualization (equal); funding acquisition (equal); investigation (equal); methodology (equal); supervision (equal); writing – review and editing (equal). **Brent K. Hollenbeck:** Conceptualization (equal); funding acquisition (equal); methodology (equal); resources (equal); supervision (equal); writing – review and editing (equal).

## FUNDING INFORMATION

Avinash Maganty is supported by funding from the National Cancer Institute Postdoctoral Fellow Award F32 Grant F32 CA275021‐01. This work is also supported by a Research Scholar Grant from the American Cancer Society (RSGI‐21‐097‐01‐HOPS).

## CONFLICT OF INTEREST STATEMENT

All authors have no disclosures or conflicts of interest to report.

## ETHICS STATEMENT

The study was deemed exempt from review by the University of Michigan institutional review board.

## Data Availability

This study used Medicare claims data, provided by the Centers for Medicare & Medicaid Services (CMS) under license/by permission. Data may be shared on request to the corresponding author with permission of the CMS.
